# The association between coffee consumption and bladder cancer in the bladder cancer epidemiology and nutritional determinants (BLEND) international pooled study

**DOI:** 10.1007/s10552-019-01191-1

**Published:** 2019-05-30

**Authors:** Evan Yi-Wen Yu, Anke Wesselius, Frits van Osch, Mariana Carla Stern, Xuejuan Jiang, Eliane Kellen, Chih-Ming Lu, Hermann Pohlabeln, Gunnar Steineck, James Marshall, Mohamed Farouk Allam, Carlo La Vecchia, Kenneth C. Johnson, Simone Benhamou, Zuo-Feng Zhang, Cristina Bosetti, Jack A. Taylor, Maurice P. Zeegers

**Affiliations:** 1 NUTRIM School for Nutrition and Translational Research in Metabolism, University of Maastricht, Universiteitssingel 40 (Room C5.564), 6229 ER Maastricht, The Netherlands; 2 CAPHRI School for Public Health and Primary Care, University of Maastricht, Maastricht, The Netherlands; 3 Institute of Cancer and Genomic Sciences, University of Birmingham, Birmingham, UK; 4 Department of Preventive Medicine, University of Southern California, Los Angeles, CA, USA; 5 Leuven University Centre for Cancer Prevention (LUCK), Louvain, Belgium; 6 Department of Urology, Buddhist Dalin Tzu Chi General Hospital, Dalin Township, Chiayi County, Taiwan; 7 Leibniz Institute for Prevention Research and Epidemiology-BIPS, Bremen, Germany; 8 Clinical Cancer Epidemiology, Department of Oncology, Clinical Sciences, Sahlgrenska Academy, University of Gothenburg, Gothenburg, Sweden; 9 Department of Cancer Prevention and Control, Roswell Park Cancer Institute, Buffalo, NY, USA; 10 Department of Medical and Surgical Specialties, Radiological Sciences and Public Health, Section of Public Health and Human Sciences, University of Brescia, Brescia, Italy; 11 Department of Clinical Medicine and Community Health, University of Milan, Milan, Italy; 12 Department of Epidemiology and Community Medicine, University of Ottawa, Ottawa, ON, Canada; 13 INSERM U946, Variabilite Genetique et Maladies Humaines, Fondation Jean Dausset/CEPH, Paris, France; 14 Departments of Epidemiology, UCLA Center for Environmental Genomics, Fielding School of Public Health, University of California, Los Angeles (UCLA), Los Angeles, CA, USA; 15 Laboratory of Oncology, Istituto di Ricerche Farmacologiche “Mario Negri” IRCCS, Milan, Italy; 16 Epidemiology Branch and Epigenetic and Stem Cell Biology Laboratory, National Institute of Environmental Health Sciences, NIH, Research Triangle Park, NC, USA

**Keywords:** Bladder cancer, Coffee consumption, Smoking, Dose–response analyses, Population-attributable risk

## Abstract

**Background:**

Inconsistent results for coffee consumption and bladder cancer (BC) risk have been shown in epidemiological studies. This research aims to increase the understanding of the association between coffee consumption and BC risk by bringing together worldwide case–control studies on this topic.

**Methods:**

Data were collected from 13 case–control comprising of 5,911 cases and 16,172 controls. Pooled multivariate odds ratios (ORs), with corresponding 95% confidence intervals (CIs), were obtained using multilevel logistic regression models. Furthermore, linear dose–response relationships were examined using fractional polynomial models.

**Results:**

No association of BC risk was observed with coffee consumption among smokers. However, after adjustment for age, gender, and smoking, the risk was significantly increased for never smokers (ever vs. never coffee consumers: OR_model2_ 1.30, 95% CI 1.06–1.59; heavy (> 4 cups/day) coffee consumers vs. never coffee consumers: OR_model2_ 1.52, 95% CI 1.18–1.97, *p* trend = 0.23). In addition, dose–response analyses, in both the overall population and among never smokers, also showed a significant increased BC risk for coffee consumption of more than four cups per day. Among smokers, a significant increased BC risk was shown only after consumption of more than six cups per day.

**Conclusion:**

This research suggests that positive associations between coffee consumption and BC among never smokers but not smokers.

## Introduction

Bladder cancer (BC) is the most common malignancy of the urinary tract and the seventh cause of death from cancer (2.8% of all cancer deaths), with nearly 430,000 new diagnoses and 165,000 deaths per year worldwide [1, 2]. Three-quarters of all BC cases occur in men [3], and most BC cases occur in the United States, Canada, and the European Union [4–7]. As with many solid tumors, BC incidence increases with age and it rarely occurs before the age of 40–50 years [8]. Given that this cancer is easy to relapse, BC is reported to be the most expensive life-time treatment of all cancers raging from € 80,000 to € 160,000 per patient [9]. The strongest risk factors for BC occurrence, such as tobacco smoking and harmful chemicals [10], have long been identified. However, as the bladder is an excretory organ, the role of fluid consumption in the development of BC could also be important.

Coffee is one of the most consumed beverages in the world. Since early 1970s, the possible relationship between coffee consumption and BC has been of considerable interest, when Cole et al. [11] suggested for the first time that coffee was a potential risk factor for BC. The International Agency for Research on Cancer (IARC) Monographs Programme in 1991 stated that there was limited evidence on the effect of coffee consumption on the BC risk and subsequently classified coffee as “possibly carcinogenic” (*group 2B; Monographs Volume 51*) [12]. Since then, several epidemiological studies focused on the relationship between coffee consumption and BC, however, results remained inconclusive [13–27]. In May 2016, a subsequent IARC Working Group of 23 scientists from 10 countries met to evaluate the carcinogenicity of drinking coffee and concluded that: “no consistent evidence of an association with drinking coffee, or of an exposure–response gradient”. This conclusion was based on evidence from 10 cohort studies and several population-based case–control studies conducted in Europe, the United States, and Japan [28]. An explanation for this inconsistency may be that previous studies on the relation between coffee consumption and BC risk lacked sufficient sample size to identify a significant association. In addition, since heavy coffee consumption is shown to be strongly associated with tobacco smoking [29], positive associations reported in some studies could possibly have been due to inadequate control for tobacco smoking. Moreover, several studies showed smoking to be interactive with caffeine in coffee [30–35], and, thereby, lead to misleading results in un-stratified analysis on the relation between coffee consumption and BC risk.

The present study aims to update the understanding and find more conclusive answers on the influence of coffee consumption on the BC risk by bringing together available case–control studies on the topic including almost 6,000 BC cases.

## Methods

### Study population

Data were derived from the BLadder cancer Epidemiology and Nutritional Determinants study (BLEND). BLEND is a large international epidemiology consortium aimed to pool data from available epidemiological studies on diet and BC. For the present study, 13 case–control studies (including 5,911 cases/16,172 controls), originated from nine different countries in three continents (i.e., Europe, North America, and Asia) had sufficient information on coffee consumption to be eligible for inclusion. BC cases were diagnosed and histologically confirmed through each study center of the included individual studies, with International Classification of Diseases (ICD) nine or ten. Most of the BC cases were identified in 1990s.

### Data collection and coding

Details on the methodology of the BLEND consortium have been described elsewhere [36]. Taking into account the local context of the included studies, different dietary assessment methods were adopted: (1) self-administrated food frequency questionnaire (FFQ) were used in Germany-1 [37], USA-2 [38], Canada-1 [39], France-1 [40], USA-3 [41], USA-4 [42]; (2) FFQ administered by a trained interviewer were used in USA-1 [43], Belgium-1 [44], China-1 [45], Sweden-1 [46], Spain-1 [47], Italy-1 [48], Italy-2 [49]. Coffee consumption was categorized using the hierarchal Eurocode 2 food coding system developed by the European Union [50]. To obtain unified consumption across studies, weekly, monthly, or yearly coffee cups were converted to daily cups of coffee consumption.

In addition to information on coffee and other dietary intake data, the BLEND dataset also included data on: study characteristics (design, method of dietary assessment, recall time of dietary consumption, and geographical region), participant demographics (age and gender), smoking status, and smoking pack-years.

### Statistical analyses

To assess the influence of coffee consumption on the BC risk, multilevel logistic regression analyses were used to estimate the pooled odds ratios (ORs) and 95% confidence intervals (CIs). Coffee consumption was expressed as: (1) ever (individuals who drank coffee > 0 cup/day) or never consumption; (2) based on the available data, coffee consumption was divided into six categories: never, 0–1 cup/day, 1–2 cups/day, 2–3 cups/day, 3–4 cups/day, and more than four cups/day; (3) caffeinated or decaffeinated coffee consumption. In addition, standardized analysis on coffee cup size was performed. For this we transformed a United States (U.S.) cup size to a 237 ml coffee cup size according to U.S. Food and Drug Administration [51] and an Asian cup size to a 500 ml cup size according to the questionnaire used in the study of Lu et al. [45].

The logistic regression models used never coffee consumers as the reference group and were computed as “crude model” (model 1), adjusted for age, gender, smoking (model 2), or fully adjustment (model 3) additionally adjusted other fluid consumption (i.e., water, liquid milk, tea, alcohol, carbonated drink, and juice). Smoking was defined as: 0 (never smokers); 1 [current light smokers (i.e., smoking less than 20 pack-years)]; 2 (current heavy smokers (i.e., smoking more than 20 pack-years)); 3 (former light smokers (i.e., smokers who ceased smoking over 1 year and smoked less than 20 pack-years)]; 4 [former heavy smokers [i.e., smokers who ceased smoking over 1 year and smoked more than 20 pack-years)]. In addition, the effect of ever versus never coffee consumption was also assessed using a meta-analysis approach; for this, pooled ORs (PORs) and 95% CIs were calculated by using a random-effect model stratified by geographical regions (i.e., Europe, North America and Asia) and study designs (i.e., hospital-based case–control studies and population-based case–control studies). Due to the lack of data, the influence of caffeine on BC risk was only assessed by comparing ever (caffeinated vs. decaffeinated) versus never coffee consumers based on multilevel logistic regression (model 2). To understand the relevance of the effect modification, the interaction terms between coffee consumption and age, gender, smoking status with pack-years were added to the model 2. *P* interaction < 0.10 was considered statistically significant where upon analyses were stratified for the covariate of interest to understand the relevance of the effect modification.

In our secondary analysis, a potential dose–response relationship between coffee consumption and BC was assessed by using fractional polynomial regression, in which the best fitting second order fractional polynomial regression model was defined as the model with the lowest deviance [52, 53]. A likelihood ratio test was used to assess the difference between the nonlinear and linear models to test for nonlinearity [54]. The results of the dose–response analyses were presented for each one coffee cup/day increment up to ten cups/day with stratification by smoking status (i.e., ever smokers and never smokers) and (i.e., hospital-based case–control studies and population-based case–control studies). Adjustments (model 2) were made for age, gender, and smoking (in overall population).

Finally, the population-attributable risk (PAR) of heavy coffee consumption (i.e., > 4 cups/day) on BC risk was estimated for Europe and North America, using the pooled risk estimates and the proportion of BC incidence in the population of interest.

All statistical analyses were performed with *STATA* version 14 SE (Stata Corporation, Texas, USA). *P* values below 0.05 were considered statistically significant.

## Results

### Baseline characteristics

The baseline characteristics of the study population are shown in Table 1. Of more than 22,000 participants, 5,911 cases of BC (4,639 men, 1,272 women) were identified. The median age at baseline was 61.4 years for cases and 57.2 years for controls, respectively. Approximately 35% of participants reported drinking coffee more than four cups per day, with an average consumption of four cups/day overall. At baseline, a higher coffee consumption was observed among smokers (five cups/day) compared to never smokers (three cups/day). In addition, coffee consumption showed strong interaction with smoking status as well as pack-years (*p* interaction: 0.001 and 0.042, respectively), while not interaction was found with age (*p* interaction: 0.17) and gender (*p* interaction: 0.16).

### Associations between coffee consumption and BC risk

#### Ever versus never coffee consumption

**Fig. 1:**
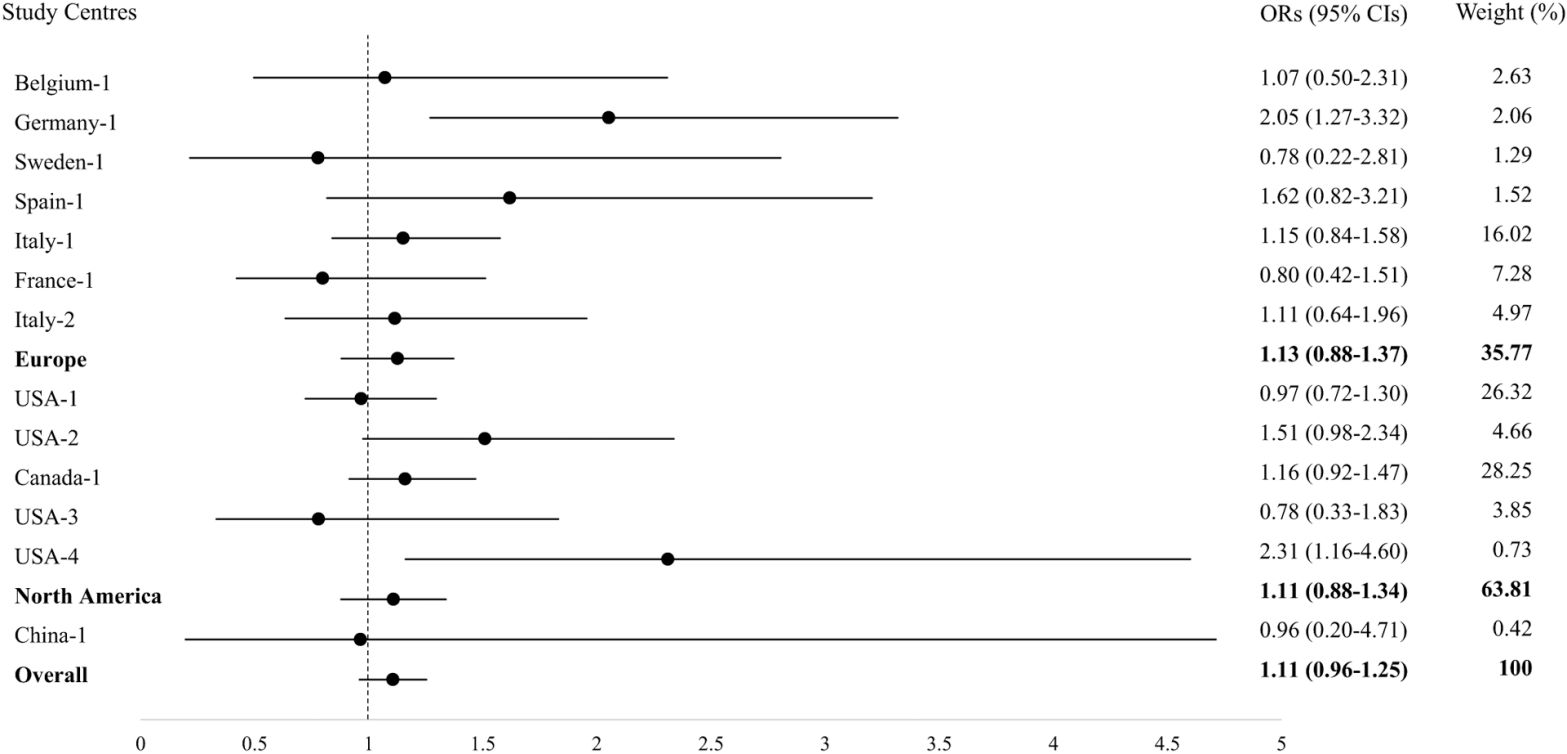
Forest Plot of Meta-Analysis with ORs and 95% CIs for Ever and Never Coffee Consumption with Bladder Cancer Risk Adjusted for Age, Gender and Smoking by Geographical Regions. *OR* odds ratio, *CI* confidence interval. Circle dots denote the odds ratios (ORs); Horizontal lines represent the 95% confidence intervals (CIs); Weights are from random-effect model. *Europe* pooled OR of studies from Europe; *North America* pooled OR of studies from North America; *Overall* pooled OR of all studies

The results comparing ever versus never coffee consumers are shown in Table 2 and Fig. 1. Overall, after adjustment for possible confounders, no statistically significant difference in BC risk could be observed between coffee consumers versus never coffee consumers (OR_model2_ 1.11, 95% CI 0.98–1.26; OR_model3_ 1.09, 95% CI 0.94–1.25). Among never smokers, a statistically significant association between coffee consumption and the risk of BC was found after further adjustment (OR_model2_ 1.30, 95% CI 1.06–1.59; OR_model3_ 1.31, 95% CI 1.03–1.66). For smokers, no significant association was observed comparing ever versus never coffee consumers. However, the estimates for former light smokers showed to be slightly higher than the estimates for other smokers. In addition, the meta-analysis stratified by geographical regions presented similar PORs based on model 2 (POR_Overall_ 1.11, 95% CI 0.96–1.25; POR_Europe_ 1.13, 95% CI 0.88–1.37; POR_North America_ 1.11, 95% CI 0.88–1.34); with low heterogeneity (*I*^2^ = 0.0%; *p* = 0.57).

#### Categories of coffee consumption with BC risk

The results of multilevel logistic regressions for subsequent categories of coffee consumption are shown in Table 2. Overall, coffee consumption of more than four cups/day results in an increased BC risk of 1.27 (95% CI 1.11–1.46, *p* trend = 0.05, model 2) compared to never coffee consumers. A similar increased risk was observed among never smokers when comparing high (> 4 cups/day) coffee consumption to never coffee consumers (OR_model2_ 1.52, 95% CI 1.19–1.94, *p* trend = 0.23). Among smokers no significant association could be observed; however, former light smokers showed again slightly higher and borderline significant results compared to other type of smokers (OR_model2_ 1.41, 95% CI 0.99–2.02, *p* trend = 0.06). The coffee cup size standardized analysis also showed significantly increased BC risk estimates with more than four cups/day coffee consumption (Supplementary Table 3).

Results for the comparison of caffeinated and decaffeinated coffee are shown in Supplementary Tables 1 and 2. For the analysis on caffeinated coffee consumption, only 1 (France-1) out of the 13 included case–control studies could be included (including 187 cases/296 controls). For the analysis on decaffeinated coffee, two studies (Italy-1 and USA-4) had sufficient data to be included in our analyses (including 1,048 cases/1,487 controls). Consumers of caffeinated coffee showed significant increased risks (compared to decaffeinated coffee consumers: OR_model2_ 1.88, 95% CI 1.42–2.48; compared to never coffee consumers: OR_model2_ 1.52, 95% CI 1.06–2.21), whereas, decaffeinated coffee consumers showed a null association with BC compared to never coffee consumers.

### Dose–response analyses

**Fig. 2:**
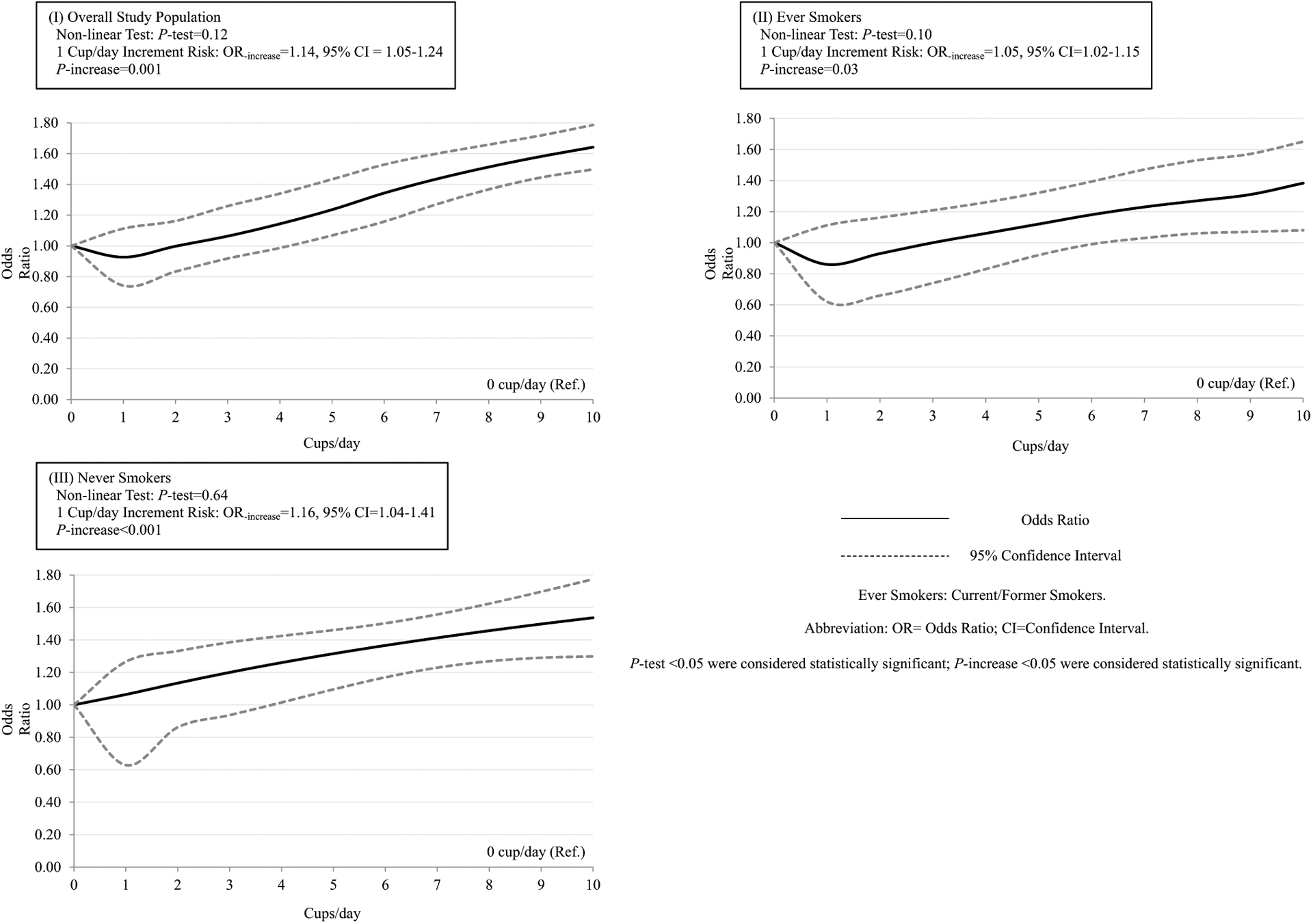
Dose-response Relationships between Coffee Consumption and the Risk of Bladder Cancer among I) Overall study population; II) Ever Smokers; III) Never Smokers. The solid lines represent the odds ratios (ORs). The dashed lines represent the 95% confidence intervals (CIs) for the trend. The ORs were adjusted for age, gender and smoking (in the overall study population) (model 2). *OR* odds ratio; *CI* confidence interval. *P* test < 0.05 were considered statistically significant; *p* increase < 0.05 were considered statistically significant

Dose–response relationships between coffee consumption and the risk of BC are displayed in Fig. 2. The tests for nonlinearity were not statistically significant; hence, linear models were applied in the dose–response analyses. The curves for the overall population showed a slightly increased BC risks with the increment of coffee consumption. Similar results were found among ever and never smokers; however, among never smokers the increased BC risk was significant for consuming over four cups/day, while for ever smokers for consumption over six cups/day. Adjusted ORs and 95% CIs for one cup/day increment were 1.14 (95% CI 1.05–1.24) in overall study population, 1.05 (95% CI 1.02–1.15) in ever smokers, 1.16 (95% CI 1.04–1.41) in never smokers. In addition, both curves for hospital-based studies and population-based studies showed increased BC risks with the increment of cups of coffee consumption per day. However, in hospital-based studies a statistically significant increase BC risk was observed for consuming over two cups/day, while for population-based studies a significant increase was observed for consuming over five cups/day (Supplementary Fig. 2).

### Population-attributable risks

Assuming an incidence of heavy coffee drinking of 28% and 17%, in Europe and North America respectively, a PAR of 7.94% for Europe and 4.45% for North America was observed.

## Discussion

This large multi centric study found an overall increased risk of BC with high (> 4 cups/day) coffee consumption. In addition, we showed that this increased risk was only observed among never smokers, but not found among smokers.

The interaction between coffee and smoking has already been studied with some detail, and experimental studies showed that smokers eliminate caffeine faster, suggesting that the effect of coffee consumption on BC risk is lower among smokers [33, 55]. In fact, faster metabolism of caffeine in smokers would allow them to consume higher levels before experiencing symptoms of caffeine toxicity [56–58]. Moreover, this hypothesis is strengthened by experimental studies reporting that the cytochrome P450 1A2 (CYP1A2) metabolic pathway is upregulated by both caffeine and compounds in tobacco smoke, including nicotine and polycyclic aromatic hydrocarbons (PAHs) [59–61], so that the effect of caffeine is potentially weaker among smokers than among never smokers. These experimental studies are in line with our result showing an increased BC risk among never smokers only. Several epidemiological studies (both case–control and cohort) also suggested a higher BC risks for coffee consumers among never smokers only or a null association among smokers [40, 62–66]. A meta-analysis of epidemiological studies also suggested that the increased BC risk of coffee consumption was higher among never smokers than among smokers [31]. However, a more recent prospective cohort study (2017) [67] conducted in the United States, which showed that high coffee consumption (> 4 cups/day) was positively associated with BC risk, could not observe an increased BC risk among never smokers only. This discrepancy may be due to the limited number of cases in their smoking-stratified analysis.

Coffee contains high content of caffeine, which has shown mutagenic effects in human cells [68], a proven influence on suppressing the activation of the protein kinase ataxia-telangiectasia mutated (ATM) and the phosphorylation of the kinase Chk2, both important for the activation of the tumor suppressor gene P53 [69, 70]. It could, therefore, be suggested that the increased BC risk is due to the caffeine content of coffee. Previous epidemiological studies already confirmed this hypothesis by showing a null association between BC risk and decaffeinated coffee [71] and an increased BC risk for caffeinated coffee [72, 73]. The present study also showed an increased BC risk when comparing caffeinated coffee consumers versus decaffeinated coffee consumers. However, due to lack of data, we were unable to perform further analyses, i.e., the association between the intensity of caffeinated coffee consumption and BC.

Besides caffeine, coffee also included other compounds, such as several phenolic compounds (i.e., chlorogenic, caffeic acid, ferulic, and coumaric acids), melanoidins, and diterpenes (i.e., cafestol and kahweol), with anti-carcinogenic properties [74–80]. This might explain why the consumption of less than four coffee cups/day showed a null association with BC risk. In addition, experimental research on the mechanisms of action of coffee compounds on P53 suppression showed this effect to be concentration dependent [69, 81–84].

The present study found a four cups/day threshold for an increased BC risk in both the overall study population and among never smokers. This finding is in line with previous reported meta-analyses based on case–control studies, also showing an increased BC risk over four cups/day (respectively OR 1.29, 95% CI 1.12–1.48; OR 1.20, 95% CI 1.12–1.24) [31, 85]. Among smokers, however, a threshold of six cups/day was observed; this again shows that the effect of coffee on BC risk might be influenced by the faster caffeine metabolism among smokers.

An issue that may arise in evaluating the influence of coffee consumption on BC risk in hospital-based case–control studies is that the controls includes patients suffering from a disease that may influence coffee intake (i.e., heart issues), resulting in inflated ORs. In the present study, therefore, stratified analyses by study design (i.e., hospital-based controls or population-based controls) were performed, and the association with coffee consumption was higher, though not significantly, in hospital-based studies than it was in population-based studies, where similar results were found in both ever versus never coffee consumption and dose–response analyses (Supplementary Figs. 1 and 2).

For the present study, the estimates of PAR showed that in Europe 7.94% and in North America 4.45% of incidence of BC cases could be attributed to heavy coffee consumption (i.e., > 4 cups/day). Unfortunately, the exact prevalence of heavy coffee drinking in the Eastern Asian population is yet unknown. However, since anecdotal evidence suggests that coffee consuming is on the rise in Eastern Asia, the PAR is expected to increase Eastern Asian countries over the next years.

Among the strengths of the BLEND study there is the large sample size, allowing to perform detailed analyses with enough statistical power to detect smaller effects, the study has also some limitations. First, it is known that the size of standard coffee cups is varied around the world, and the effect of a cup of coffee on BC might, therefore, differ between different countries. However, our per-center analyses as well our standardized analysis, in which we transformed a United States (U.S.) cup size to a 237 ml coffee cup size according to U.S. Food and Drug Administration [51] and an Asian cup size to a 500 ml cup size according to the questionnaire used in the study of Lu et al. [45], showed similar results (Supplementary Table 3: OR_model2_ 1.25, 95% CI 1.06–1.47, *p* trend = 0.18). Due to large heterogeneity among coffee cup size among European countries, Europe was not included in these standardized analyses. In addition, it is suggested that the strength of coffee brew may compensate for the different serving size between countries [86].

Second, it is often suggested that case–control studies are limited in showing causal relation, due to the potential recall bias of case–control studies. This might have led to a lower reliability of the results compared to those of cohort studies. However, although this issue has been addressed and analyzed for its consequences in many epidemiological/methodological papers [87–90], no clear answer on the magnitude of the effect of this specific type of bias could be draw.

Thirdly, limited information was available on possible risk factors, other than age, gender and smoking, for the development of BC, such as body mass index (BMI), physical activity, socioeconomic status (SES), disinfection byproducts, arsenic in the drinking water, and occupational exposures to potentially carcinogenic chemicals. Although, adjustments for these factors could have influenced the results, current literature shows that only a small proportion of BC cases can be attributed to these factors [72]. Last, although status as well as duration and intensity of smoking were taken into account in our analysis, the adjustment for smoking might still be imperfect due to differences in smoking practices (e.g., depth of inhalation or amount of inhalation), or differences in types of smoke exposure [67]. In addition, since smoking is perceived as a socially undesirable behavior, the use of self-reported questionnaires for smoking status, duration, and intensity might have led to underreporting of the actual smoking habits.

## Conclusion

In summary, the present study, with more than 5,900 cases, observed an increased risk between high (> 4 cups/day) coffee consumption and BC among never smokers, while no association with BC risk was observed with coffee consumption among smokers. Additionally, it indicates that around 7.94% of BC cases for Europe and 4.45% of BC cases for North America in the population might be attributable to heavy coffee consumption (> 4 cups/day).

## Supplementary Material

Supplementary Material

## Figures and Tables

**Table 1:** Characteristics of the study population (5,911 cases and 16,172 controls) and coffee consumption category according to age, gender and smoking status

Characteristics	Coffee consumption								Mean (± SD) cups/day	*p* value^a^	*p* interaction
	Total	Never	Ever	≤ 1 cups/day	1–2 cups/day	2–3 cups/day	3–4 cups/day	> 4 cups/day			
*n (%)*	22,083 (100)	2,372 (10.74)	19,711 (89.26)	2,752 (12.46)	3,616 (16.37)	2,962 (13.41)	2,750 (12.45)	7,631 (34.56)	4.00 (±3.97)	< 0.001	
Case (%)	5,911 (100)	499 (8.44)	5,412 (91.56)	659 (11.15)	1,077 (18.22)	716 (12.11)	801 (13.55)	2,159 (36.53)	4.58 (±4.84)		
Control (%)	16,172 (100)	1,873 (11.58)	14,299 (88.42)	2,093 (12.94)	2,539 (15.70)	2,246 (13.89)	1,949 (12.05)	5,472 (33.84)	3.78 (±3.58)		
Gender											0.16
Male (%)											
Case	4,639 (100)	369 (7.95)	4,270 (92.05)	488 (10.52)	865 (18.65)	560 (12.07)	646 (13.93)	1,711 (36.88)	4.70 (±4.99)	< 0.001	
Control	8,983 (100)	1,002 (11.15)	7,981 (88.85)	1,240 (13.80)	1,468 (16.34)	1,240 (13.80)	1,114 (12.40)	2,919 (32.49)	3.84 (±3.98)		
Female (%)											
Case	1,272 (100)	130 (10.22)	1,142 (89.78)	171 (13.44)	212 (16.67)	156 (12.26)	155 (12.19)	448 (35.22)	4.13 (±4.21)	0.06	
Control	7,189 (100)	871 (12.12)	6,318 (87.88)	853 (11.87)	1,071 (14.90)	1,006 (13.99)	835 (11.61)	2,553 (35.51)	3.71 (±3.01)		
Age (Mean ± SD)											
Case	5,911 (100)	60.48 (± 12.70)	5, 412 (91.56)	65.38 (±9.88)	64.36 (± 10.36)	64.34 (±8.65)	61.61 (±9.98)	59.88 (±9.21)	4.52 (±4.82)	< 0.001	0.17
Control	16,172 (100)	59.92 (±8.49)	14,299 (88.42)	61.18 (±8.52)	61.94 (±8.80)	61.57 (±8.15)	60.52 (±9.32)	58.92 (±10.13)	3.80 (±3.59)		
Smoking status											
Current smokers (%)											0.001
Case	2,317 (100)	140 (6.04)	2,177 (93.96)	176 (7.60)	417 (18.00)	258 (11.14)	359 (15.49)	967 (41.74)	4.95 (±4.60)	< 0.001	
Control	5,350 (100)	357 (6.67)	4,993 (93.33)	324 (6.06)	782 (14.62)	478 (8.93)	751 (14.04)	2,658 (49.68)	4.92 (±3.75)		
Former smokers (%)											
Case	2,491 (100)	187 (7.51)	2,304 (92.49)	310 (12.44)	412 (16.54)	356 (14.29)	298 (11.96)	928 (37.25)	4.88 (±5.50)	< 0.001	
Control	4,636 (100)	464 (10.01)	4,172 (89.99)	845 (18.23)	637 (13.74)	946 (20.41)	415 (8.95)	1,329 (28.67)	3.49 (±3.92)		
Never smokers (%)											
Case	1,103 (100)	172 (15.59)	931 (84.41)	173 (15.68)	248 (22.48)	102 (9.25)	144 (13.06)	264 (23.93)	3.11 (±3.14)	< 0.001	
Control	6,186 (100)	1,052 (17.01)	5,134 (82.99)	924 (14.94)	1,120 (18.11)	822 (13.29)	783 (12.66)	1, 485 (24.01)	3.01 (±2.84)		

*n* number, *SD* standard deviation

a Calculated by *χ*^2^ test for categorical variables and *t* test for continuous variables

*p* values < 0.05 were considered statistically significant

*p* interaction < 0.10 were considered statistically significant

**Table 2:** Adjusted odds ratios and 95% confidence intervals of bladder cancer according to coffee consumption level stratified by smoking

Study sub-groups	Model adjustments	Coffee consumption (ORs and 95% CI)							*p* trend
		Never	Ever	≤ 1 cups/day	1–2 cups/day	2–3 cups/day	3–4 cups/day	> 4 cups/day	
Overall (*n* = 22,083)	Model 1^a^	Reference	1.42 (1.26–1.59)	1.05 (0.91–1.22)	1.35 (1.17–1.55)	1.23 (1.06–1.42)	1.51 (1.31–1.76)	1.82 (1.60–2.07)	0.04
		
	Model 2^b^	Reference	1.11 (0.98–1.26)	0.94 (0.80–1.10)	1.12 (0.96–1.29)	1.01 (0.86–1.17)	1.16 (0.99–1.35)	1.27 (1.11–1.46)	0.05
			
	Model 3^c^	Reference	1.09 (0.94–1.25)	0.92 (0.77–1.10)	1.04 (0.88–1.24)	0.97 (0.81–1.15)	1.15 (0.96–1.37)	1.24 (1.06–1.45)	0.11
			
Current light smokers (*n* = 3,548)	Model 1^a^	Reference	1.01 (0.68–1.47)	0.75 (0.43–1.30)	0.99 (0.64–1.53)	0.71 (0.42–1.19)	1.31 (0.84–2.04)	1.09 (0.72–1.63)	0.34
		
	Model 2^b^	Reference	0.97 (0.65–1.43)	0.63 (0.36–1.11)	0.92 (0.59–1.44)	0.71 (0.42–−1.21)	1.24 (0.79–1.94)	1.09 (0.72–1.66)	0.23
			
	Model 3^c^	Reference	1.16 (0.71–1.90)	0.71 (0.37–1.35)	1.09 (0.63–1.88)	0.80 (0.43–1.49)	1.46 (0.85–2.48)	1.25 (0.78–2.07)	0.14
			
Current heavy smokers (*n* = 3,458)	Model 1^a^	Reference	1.01 (0.71–1.41)	0.91 (0.60–1.39)	1.08 (0.74–1.60)	0.92 (0.63–1.35)	0.97 (0.65–1.44)	1.06 (0.74–1.51)	0.66
		
	Model 2^b^	Reference	1.01 (0.71–1.42)	0.83 (0.54–1.28)	0.99 (0.67–1.47)	0.91 (0.62–1.35)	0.97 (0.65–1.46)	1.15 (0.80–1.66)	0.57
			
	Model 3^c^	Reference	0.92 (0.59–1.41)	0.75 (0.45–1.24)	0.78 (0.48–1.29)	0.82 (0.51–1.32)	0.89 (0.55–1.45)	1.03 (0.66–1.61)	0.34
			
Former light smokers (*n* = 3,314)	Model 1^a^	Reference	1.24 (0.91–1.70)	1.03 (0.71–1.51)	1.20 (0.82–1.76)	1.36 (0.95–1.97)	1.37 (0.90–2.08)	1.29 (0.91–1.83)	0.07
		
	Model 2^b^	Reference	1.28 (0.93–1.75)	1.01 (0.68–1.48)	1.19 (0.81–1.75)	1.44 (0.99–2.09)	1.36 (0.89–2.08)	1.41 (0.99–2.02)	0.06
			
	Model 3^c^	Reference	1.30 (0.90–1.88)	1.05 (0.68–1.62)	1.14 (0.74–1.77)	1.50 (0.99–2.27)	1.39 (0.87–2.21)	1.38 (0.93–2.07)	0.09
			
Former heavy smokers (*n* = 3,202)	Model 1^a^	Reference	1.02 (0.76–1.37)	0.83 (0.58–1.17)	0.96 (0.67–1.37)	0.90 (0.64–1.26)	1.03 (0.71–1.49)	1.28 (0.93–1.76)	0.19
		
	Model 2^b^	Reference	1.01 (0.75–1.35)	0.80 (0.57–1.13)	0.93 (0.65–1.33)	0.89 (0.63–1.24)	1.03 (0.71–1.49)	1.28 (0.93–1.76)	0.18
			
	Model 3^c^	Reference	0.92 (0.66–1.27)	0.72 (0.49–1.05)	0.79 (0.53–1.18)	0.78 (0.54–1.13)	0.96 (0.64–1.44)	1.20 (0.85–1.70)	0.07
			
Never smokers (*n* = 7,289)	Model 1^a^	Reference	1.36 (1.12–1.67)	1.31 (1.01–1.70)	1.53 (1.20–1.96)	1.04 (0.78–1.39)	1.31 (0.99–1.74)	1.52 (1.19–1.94)	0.23
		
	Model 2^b^	Reference	1.30 (1.06–1.59)	1.18 (0.90–1.55)	1.36 (1.05–1.76)	0.96 (0.71–1.30)	1.22 (0.91–1.64)	1.52 (1.18–1.97)	0.23
			
	Model 3^c^	Reference	1.31 (1.03–1.66)	1.28 (0.95–1.73)	1.42 (1.06–1.89)	0.97 (0.70–1.34)	1.24 (0.91–1.70)	1.51 (1.15–1.99)	0.24
			

Referent group was never coffee consumers

*OR* odds ratio, *CI* confidence interval

a Model 1: Crude model

b Model 2: Adjusted for age, gender and smoking (in the overall analyses)

c Model 3: Additionally adjusted for water, liquid milk, alcohol, carbonated drinks, tea and juice

*p* trend < 0.05 were considered statistically significant

## Abbreviations

BC: Bladder cancer
IARC: International Agency for Research on Cancer
BLEND: Bladder cancer epidemiology and nutritional determinants
FFQ: Food frequency questionnaires
OR: Odds ratio
CI: Confidence interval
POR: Pooled odds ratio
CYP1A2: Cytochrome P450 1A2
PAHs: Polycyclic aromatic hydrocarbons
ATM: Ataxia-telangiectasia mutated
PAR: Population-attributable risk
BMI: Body mass index
SES: Socioeconomic status
