# Supplementary material for: The association between coffee consumption and bladder cancer in the bladder cancer epidemiology and nutritional determinants (BLEND) international pooled study
Source: Cancer Causes Control. Author manuscript; Available in PMC 2022 Apr 6. (PMC8985651; doi:10.1007/s10552-019-01191-1)
Supplement: Supplementary Material [file NIHMS1615821-supplement-Supplementary_Material.docx]

**Supplementary Materials to “The association between coffee consumption and bladder cancer in the bladder cancer epidemiology and nutritional determinants (BLEND) international pooled study”**

Evan Yi-Wen YU^*^, Anke Wesselius, Frits van Osch, Mariana Carla Stern, Xuejuan Jiang, Eliane Kellen, Chih-Ming Lu, Hermann Pohlabeln, Gunnar Steineck, James Marshall, Mohamed Farouk Allam, Carlo La Vecchia, Kenneth C. Johnson, Simone Benhamou, Zuo-Feng Zhang, Cristina Bosetti, Jack A. Taylor, Maurice P. Zeegers

**Supplementary Table 1.** Characteristics of the Study Population (1,235 Cases and 1,783 Controls) and Caffeinated or Decaffeinated Coffee Consumption According to Age, Gender and Smoking Status

**Supplementary Table 2.** Ever versus Never Analyses for Caffeinated and Decaffeinated Coffee with Bladder Cancer Risk

**Supplementary Table 3.** Adjusted Odds Ratios and 95% Confidence Intervals of Bladder Cancer According to Coffee Consumption in Never Smokers

**Supplementary Fig. 1.** Forest Plot of Meta-Analyses with ORs and 95% CIs for Ever and Never Coffee Consumption with Bladder Cancer Risk Adjusted for Age, Gender and Smoking

**Supplementary Fig. 2.** Dose-response Relationships between Coffee Consumption and the Risk of Bladder Cancer among Never Smokers: I) Population-based Case-control Studies; II) Hospital-based Case-controls Studies

**Supplementary Table 1.** Characteristics of the Study Population (1,235 Cases and 1,783 Controls) and Caffeinated or Decaffeinated Coffee Consumption According to Age, Gender and Smoking Status

| Characteristics |  | Coffee Consumption | | | Mean (±SD)  cups/day | *P-*value^a^ |
| --- | --- | --- | --- | --- | --- | --- |
|  |  | **Never** | **Decaffeinated coffee** | **Caffeinated coffee** |  |  |
| *N* (%) |  | 1,857 (73.23) | 247 (9.74) | 432 (17.03) | 3.69 (±4.39) | 0.001 |
| Case (%) |  | 751 (71.66) | 129 (12.31) | 168 (16.03) | 4.30 (±3.59) |  |
| Control (%) |  | 1,106 (74.33) | 118 (7.93) | 264 (17.74) | 3.28 (±4.40) |  |
| Gender |  |  |  |  |  |  |
| Male (%) | Case | 632 (72.56) | 92 (7.55) | 147 (16.88) | 4.20 (±3.65) | 0.02 |
|  | Control | 814 (72.29) | 85 (7.55) | 227 (20.16) | 3.59 (±4.69) |  |
| Female (%) | Case | 119 (67.23) | 37 (20.90) | 21 (11.86) | 4.79 (±3.26) | <0.001 |
|  | Control | 292 (80.66) | 33 (9.12) | 37 (10.22) | 2.33 (±4.34) |  |
| Age (Mean±SD) | Case | 63.16 (±8.39) | 65.84 (±8.27) | 65.43 (±11.34) | 4.30 (±3.59) | <0.001 |
|  | Control | 59.38 (±10.39) | 65.18 (±8.48) | 66.64 (±9.86) | 3.28 (±4.40) |  |
| Smoking Status |  |  |  |  |  |  |
| Current Smokers (%) | Case | 356 (77.39) | 42 (9.13) | 62 (13.48) | 4.02 (±3.08) | 0.01 |
|  | Control | 356 (84.16) | 18 (4.26) | 49 (11.58) | 2.19 (±4.96) |  |
| Former Smokers (%) | Case | 266 (64.25) | 63 (15.22) | 85 (20.53) | 4.99 (±3.76) | 0.04 |
|  | Control | 333 (64.04) | 55 (10.58) | 132 (25.38) | 4.55 (±4.30) |  |
| Never Smokers (%) | Case | 129 (74.14) | 24 (13.79) | 21 (12.07) | 3.38 (±3.72) | 0.07 |
|  | Control | 417 (76.51) | 45 (8.26) | 83 (15.23) | 2.92 (±4.62) |  |

^a^ Calculated by χ ^2^ test for categorical variables and *t* test for continuous variables.

Abbreviation: *N*=Number; SD=Standard Deviation.

*P*-values <0.05 were considered statistically significant.

**Supplementary Table 2.** Analyses for Caffeinated and Decaffeinated Coffee with Bladder Cancer Risk Adjusted for Age, Gender and Smoking

| **Study Design** | **Coffee Consumption (ORs and 95% CI)** | | |  |
| --- | --- | --- | --- | --- |
|  | **Never** | **Decaffeinated**^a^ | **Caffeinated^a^** | |
| **Caffeinated versus Decaffeinated** | — | Reference | 1.88 (1.42-2.48) | |
| **Caffeinated versus Never** | Reference | — | 1.52 (1.06-2.21) | |
| **Decaffeinated versus Never** | Reference | 0.76 (0.29-2.03) | — | |

^a^ Model 2: Adjusted for age, gender and smoking.

Abbreviation: OR=Odds Ratio; CI=Confidence Interval.

Referent groups were never coffee consumers or decaffeinated coffee consumers.

**Supplementary Table 3.** Adjusted Odds Ratios and 95% Confidence Intervals of Bladder Cancer According to Coffee Consumption in Never Smokers

| **Model Adjustments** | | **Coffee Consumption (ORs and 95% CI)** | | | | | | ***P-*trend** |
| --- | --- | --- | --- | --- | --- | --- | --- | --- |
|  |  | **Never** | **≤1 cup/day** | **1-2 cups/day** | **2-3 cups/day** | **3-4 cups/day** | **> 4 cups/day** |  |
| **Unified Cup Size**  **(237ml/cup)** | Model 1^a^ | Reference | 0.97 (0.80-1.18) | 1.40 (1.15-1.70) | 1.14 (0.95-1.38) | 1.50 (1.24-1.81) | 1.76 (1.51-2.06) | 0.05 |
|  | Model 2^b^ | Reference | 0.83 (0.68-1.02) | 1.16 (0.95-1.43) | 0.92 (0.76-1.12) | 1.14 (0.94-1.40) | 1.25 (1.06-1.47) | 0.18 |
|  | Model 3^c^ | Reference | 0.85 (0.69-1.04) | 1.12 (0.90-1.39) | 0.98 (0.80-1.20) | 1.30 (1.06-1.60) | 1.56 (1.32-1.85) | 0.11 |

^a^ Model 1: Crude model without confounders adjustment.

^b^ Model 2: Adjusted for age and gender.

^c^ Model 3: Additionally adjusted for water, liquid milk, alcohol, carbonated drinks, tea and juice.

Abbreviation: OR= Odds Ratio; CI=Confidence Interval.

Referent group was never coffee consumers.

*P*-trend <0.05 were considered statistically significant.

USA-1

Belgium-1

Sweden-1

Italy-1

Canada-1

USA-3

Italy-2

**Population-Based**

China-1

Germany-1

USA-2

Spain-1

French-1

USA-4

**Hospital-Based**

**Overall**

0.97 (0.72-1.30)

1.07 (0.50-2.31)

0.78 (0.22-2.81)

1.15 (0.84-1.58)

1.16 (0.92-1.47)

0.78 (0.33-1.83)

1.11 (0.64-1.96)

**1.07 (0.91-1.23)**

0.96 (0.20-4.71)

2.05 (1.27-3.32)

1.51 (0.98-2.34)

1.62 (0.82-3.21)

0.80 (0.42-1.51)

2.31 (1.16-4.60)

**1.41 (0.92-1.90)**

**1.11 (0.96-1.25)**

Study Centres

ORs (95% CIs)

26.32

2.63

1.29

16.02

28.25

3.85

4.97

**83.33**

0.42

2.06

4.66

1.52

7.28

0.73

**16.67**

**100**

Weight (%)

**(I^2^=0.0%; *P*=0.93)**

**(I^2^=32.30%; *P*=0.19)**

**(I^2^=0.0%; *P*=0.57)**

**Supplementary Fig. 1.** Forest Plot of Meta-Analyses with ORs and 95% CIs for Ever and Never Coffee Consumption with Bladder Cancer Risk Adjusted for Age, Gender and Smoking by Type of Control Selection

Abbreviation: OR= Odds Ratio; CI=Confidence Interval.

Circle dots denote the odds ratios (ORs); Horizontal lines represent the 95% confidence intervals (CIs); Weights are from random effects analyses.

**Population-Based**: pooled OR of population-based case-control studies; **Hospital-Based**: pooled OR of hospital-based case-control studies; **Overall**: pooled OR of all studies.

(I) Population-based Case-control Studies

Non-linear Test: *P*-test=0.87

1 Cup/day Increment Risk: OR_-increase_=1.06, 95% CI=1.03-1.08

*P*-increase<0.001

(II) Hospital-based Case-control Studies

Non-linear Test: *P*-test=0.25

1 Cup/day Increment Risk: OR_-increase_=1.13, 95% CI=1.10-1.15

*P*-increase<0.001

Abbreviation: OR= Odds Ratio; CI=Confidence Interval.

Ever Smokers: Current/Former Smokers.

Odds Ratio

Odds Ratio

Cups/day

Cups/day

Odds Ratio

95% Confidence Interval

**Supplementary Fig. 2.** Dose-response Relationships between Coffee Consumption and the Risk of Bladder Cancer among Never Smokers: I) Population-based Case-control Studies; II) Hospital-based Case-control Studies

The solid lines represent the odds ratios (ORs). The dashed lines represent the 95% confidence intervals (CIs) for the trend. The ORs were adjusted for age, gender and smoking (in the overall study population) (model 2). *P*-test <0.05 were considered statistically significant; *P*-increase <0.05 were considered statistically significant.
